# Parasite-Derived Plasma Microparticles Contribute Significantly to Malaria Infection-Induced Inflammation through Potent Macrophage Stimulation

**DOI:** 10.1371/journal.ppat.1000744

**Published:** 2010-01-29

**Authors:** Kevin N. Couper, Tom Barnes, Julius C. R. Hafalla, Valery Combes, Bernhard Ryffel, Thomas Secher, Georges E. Grau, Eleanor M. Riley, J. Brian de Souza

**Affiliations:** 1 Immunology Unit, Department of Infectious and Tropical Diseases, London School of Hygiene and Tropical Medicine, London, United Kingdom; 2 Department of Immunology and Molecular Pathology, University College London Medical School, London, United Kingdom; 3 Department of Pathology, University of Sydney, Camperdown, New South Wales, Australia; 4 Molecular Immunology and Embryology, University of Orleans and Centre National de la Recherche Scientifique, Orleans, France; Case Western Reserve University, United States of America

## Abstract

There is considerable debate as to the nature of the primary parasite-derived moieties that activate innate pro-inflammatory responses during malaria infection. Microparticles (MPs), which are produced by numerous cell types following vesiculation of the cellular membrane as a consequence of cell death or immune-activation, exert strong pro-inflammatory activity in other disease states. Here we demonstrate that MPs, derived from the plasma of malaria infected mice, but not naive mice, induce potent activation of macrophages in vitro as measured by CD40 up-regulation and TNF production. In vitro, these MPs induced significantly higher levels of macrophage activation than intact infected red blood cells. Immunofluorescence staining revealed that MPs contained significant amounts of parasite material indicating that they are derived primarily from infected red blood cells rather than platelets or endothelial cells. MP driven macrophage activation was completely abolished in the absence of MyD88 and TLR-4 signalling. Similar levels of immunogenic MPs were produced in WT and in TNF^−/−^, IFN-γ^−/−^, IL-12^−/−^ and RAG-1^−/−^ malaria-infected mice, but were not produced in mice injected with LPS, showing that inflammation is not required for the production of MPs during malaria infection. This study therefore establishes parasitized red blood cell-derived MPs as a major inducer of systemic inflammation during malaria infection, raising important questions about their role in severe disease and in the generation of adaptive immune responses.

## Author Summary

Although parasite materials are responsible for the activation of the immune system during malaria infection, exactly how the immune response is initiated during infection is extremely unclear. In this study we demonstrate that sub micron particles (microparticles) are produced by malaria infected red blood cells during malaria infection, and we show that these microparticles can promote strong inflammatory responses by activating macrophages. We show that infected red blood cell-derived microparticles are produced in higher numbers as infection progresses, and that the host's own pro-inflammatory immune response is not required for the generation of these microparticles. We have also examined the receptors and signalling pathways required for macrophage activation by microparticles, and we show that the pathway of microparticle-induced activation is distinct from other previously reported pathways. In summary, we have defined a novel pathway of immune response activation during malaria infection, which may be important for promoting parasite control and/or causing pathology.

## Introduction

Severe malaria in humans is a leading cause of morbidity and mortality, especially in sub-Saharan Africa [1]. The clinical manifestations of severe malaria are directly correlated with the induction of strong pro-inflammatory type-1 immune responses. Thus, whilst it is clear that early innate and T cell pro-inflammatory immune responses are essential for the control of malaria infection [2],[3], excessive production of pro-inflammatory cytokines, including IL-6, TNF and IFN-γ, may also directly contribute to severe disease, such as severe anaemia, cerebral malaria (CM) and organ damage [3],[4]. It is therefore crucial that the most potent parasite dependent and independent pro-inflammatory triggers are identified, and their signalling pathways unravelled, before targeted, successful therapeutic treatments can be developed for malaria infection.

Activation of macrophages is a key event in the pathogenesis of severe malaria in both humans [5] and in experimental models of malaria [6]. *P. berghei* ANKA (PbA) infection of C57BL/6 mice, which is the best available model of CM, is characterized by the development of strong pro-inflammatory immune responses, including macrophage activation and the production of TNF, IL-12, IL-1β, IL-6, ROI and NO [2],[3]. Activation of brain resident and brain-homing monocytic cells, leading to activation of brain vascular endothelial cells and consequent sequestration of pRBC and leucocytes, is believed to be a key stage in the development of the neuropathology associated with experimental cerebral malaria (ECM) during PbA infection. In addition, although splenic and liver macrophage populations have been shown to be required for optimal parasite control [7],[8], excessive macrophage responses in these organs has been directly correlated with malarial anaemia and liver damage [9].

At present there is considerable debate about the pathways driving inflammation during malaria infection. Interaction of malaria parasite-derived moieties with cells of the innate system, such as macrophages and dendritic cells, is likely to be the initial step in induction of the inflammatory response; however, despite intense research, there is no agreement regarding the identity of the primary parasite products that initiate the pro-inflammatory cascade [4] and the importance of Toll like receptor signalling and scavenger receptors (such as CD36) in the recognition of parasite products and subsequent production of inflammatory cytokines remains unclear [10]–[21]. Parasitized red blood cells (pRBC) have been shown, depending on the model and the duration of stimulation, to either induce or suppress macrophage and dendritic cell function, including induction or suppression of pro-inflammatory cytokine production [21]–[26]. The malaria pigment hemozoin has been proposed as a novel TLR-9 ligand, inducing TNF, IL-6 and IL-12 p40 production [12], but it has subsequently been suggested that contaminating malarial DNA, which binds to hemozoin, is responsible for TLR-9 activation [27]. However, hemozoin has also been shown to directly suppress macrophage and dendritic cell function [28]–[30]. In recent years attention has focussed on the potential role of parasite glycosylphosphatidyl-inositol (GPI) [19],[20],[31],[32], which is capable of inducing TNF secretion by macrophages via signalling through TLR-2 [20] and the scavenger receptor CD36 [19] and which, in one report, induced cachexia when injected into mice [31]. Most recently it has been shown that plasma-derived microparticles (MPs) from malaria-infected mice can induce TNF production by macrophages [33] suggesting that MPs may also contribute to the systemic inflammation that is characteristic of malaria infection.

MPs are sub-micron particles (0.1–1µm diameter) produced by vesiculation or ‘blebbing’ of the plasma membrane of cells as a result of loss of asymmetry of the phospholipid bilayer (reviewed [34],[35]). In healthy animals, circulating MPs are predominantly derived from platelets, but, depending on the situation, MPs may also be produced by leucocytes, endothelial cells and erythrocytes (reviewed [34],[35]). Vesiculation of the phospholipid bilayer and MP development is a tightly-regulated homeostatic process that occurs at an increased rate during cell activation and during apoptotic or necrotic cell death. MP formation is directly correlated with TNF and IL-1β production [34],[35]. Thus, although basal levels of MP are found in the blood of healthy donors, elevated levels have been detected in many pathological conditions [34],[35] including cerebral malaria [36]. Consistent with results obtained in humans, significantly higher numbers of circulating MPs are found in PbA-infected mice than in uninfected controls [33],[35].

In this study we have investigated the ability of MPs induced during acute malaria infections in mice to stimulate macrophage pro-inflammatory responses and we have assessed the potential relevance of this pathway in the development of severe malarial disease. We find that malaria infection-induced MPs are much more potent inducers of macrophage activation than are intact, live pRBC and that pRBC-derived MPs, rather than endothelial-, leukocyte- or platelet-derived MPs, are the primary inducers of macrophage activation. Furthermore, we have defined a TLR-4- and MyD88-dependent pathway of MP-induced macrophage activation. This study establishes a major new pathway of innate inflammation during malaria infection which implicates pRBC-derived MPs as major contributors to the development of severe malarial disease.

## Results

### Malaria infection-derived microparticles activate macrophages in vitro

**Figure 1:**
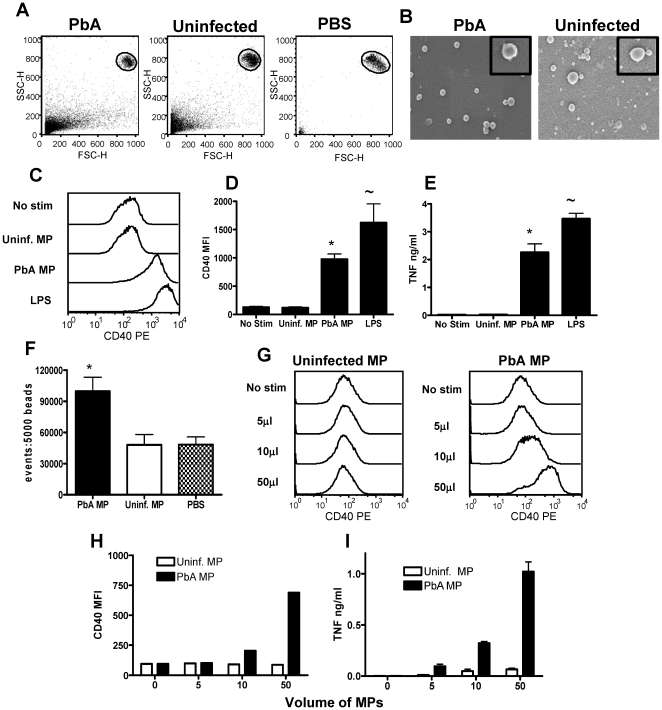
Plasma microparticles derived from malaria infected mice stimulate strong macrophage pro-inflammatory responses in vitro. Microparticles were prepared from the plasma of uninfected mice (uninfected MP) and from mice infected with *P. berghei* ANKA (day 7: PbA MP) and were used to stimulate bone-marrow derived macrophages in vitro for 24hrs. (A) The size of plasma derived microparticles relative to 1µm beads is shown. (B) Scanning electron micrographs showing morphology of MPs. Magnification 72,000X (insert 240,000X). (C) Representative histograms showing the level of CD40 expression by bone marrow derived macrophages following stimulation with uninfected MPs, PbA MPs and LPS. (D) Mean fluorescence intensity of CD40 expression by macrophages following stimulation. * p<0.05 between PbA MP and no-stim; ∼ p<0.05 between LPS and no stim (E) The level of TNF production by stimulated macrophages was measured in the supernatant by ELISA. * p<0.05 between PbA MP and no-stim; ∼ p<0.05 between LPS and no stim (F) The number of MPs within the uninfected and PbA derived preparations was calculated relative to a standardised number of 1µm beads. * p<0.05 between PbA MP and uninfected MP. (G) Representative histograms showing the expression level of CD40 on macrophages following stimulation with varying doses of (left plot) uninfected MPs and (right plot) PbA-derived MPs. (H) The mean fluorescence intensity of CD40 expression on macrophages stimulated with varying doses of uninfected and PbA-derived MPs. (I) TNF production by stimulated with varying doses of uninfected and PbA-derived MPs. The results are representative of 4 separate experiments.

To investigate the ability of *Plasmodium berghei* ANKA (PbA)-induced MPs to activate macrophages *in vitro*, bone marrow-derived macrophages were challenged for 24hrs with purified PbA infection-induced MPs, and uninfected MPs derived from naive mice; macrophage activation was assessed by up-regulation of CD40 expression and by the production of TNF. The size and granularity of the purified MP preparations relative to 1µm beads (gated population: upper right hand side) is shown in representative plots in Fig. 1A. As can be observed, PbA infection-induced MPs and uninfected MPs were sub-cellular in size and approximately 98% of all flow cytometric events within the MP preparation were <1µm in size. A number of flow cytometric events were observed in the PBS control, but these events were in general smaller than those observed in the MP preparations and were due to minor contaminations within the solution. PbA infection-induced MPs were homogenous in size and the majority of MPs were approximately 150–250nm in diameter when examined by scanning electron microscopy (Fig. 1B). Uninfected MPs were heterogeneous in size compared with PbA-infection induced MPs, varying from approximately 75µm to 450µm in diameter. Irrespective of size, uninfected MPs and PbA infection-induced MPs were comparable in morphology and appeared spherical in appearance (Fig. 1B).

As expected, incubation with PBS (no stimulation) failed to induce macrophage activation, as measured by CD40 expression and TNF production (Fig. 1C–E). Similarly, stimulation with control MPs from uninfected mice also failed to induce up-regulation of CD40 expression or the production of TNF, indicating that MPs derived from uninfected mice are non-inflammatory (Fig. 1C–E). In contrast, strong macrophage activation was observed following stimulation with PbA-induced MPs (using a comparable volume of the MP preparation as used for control MPs), with significantly elevated expression of CD40 and increased production of TNF compared with non-stimulated and uninfected MP stimulated controls (Fig. 1C–E). These data confirm the results obtained by Combes et al [33] showing that PbA-induced MPs can stimulate TNF production by macrophages. To ensure that macrophage activation was not an artefact of LPS contamination of the MPs, endotoxin concentrations were tested in all the MP preparations and were found to be less than 0.24 IU/ml in all cases; the minimum concentration of LPS required to activate macrophages is approx 0.6 IU/ml (data not shown but provided for review).

Significantly increased numbers of flow cytometric events were found within PbA infection-induced MP preparations compared with uninfected MP preparations (Fig. 1F): thus, it was foreseeable that the ability of PbA induced MPs - but not uninfected MPs - to stimulate macrophages was related to a quantitative difference in the number of inflammatory MP particles, over an unspecified threshold level, rather than an intrinsic qualitative difference in the immunogenicity of the MP preparations. To examine this we performed a dose response experiment using different volumes of PbA induced and uninfected MP preparations and examined the ability of the MPs to activate macrophages: uninfected MPs derived from naive mice failed to stimulate CD40 up-regulation or TNF production at any of the tested concentrations, whereas PbA induced MPs promoted up-regulation of CD40 expression and production of TNF in a dose dependent manner at 5, 10 and 50µl volumes, equating to approximately 5×10^4^, 1×10^5^ and 5×10^5^ PbA MPs/well respectively (Fig. 1G–I). In addition, uninfected MPs failed to stimulate macrophage activation when the number of flow cytometric events of the preparation was normalised to PbA MPs numbers (results not shown). The kinetics of macrophage activation by PbA-induced MPs or LPS were compared. Although both LPS and PbA-induced MPs induced a maximal TNF response within 6 hrs, maximal CD40 induction was slower for PbA-induced MPs (24–48 hrs) than for LPS (12hrs) (Fig. S1). Taken together, these results demonstrate that MPs derived from malaria infected mice induce potent macrophage activation and are significantly more inflammatory on a particle to particle basis than MPs from normal, uninfected mice.

### Infection derived microparticles are highly pro-inflammatory compared with parasitized red blood cells

**Figure 2:**
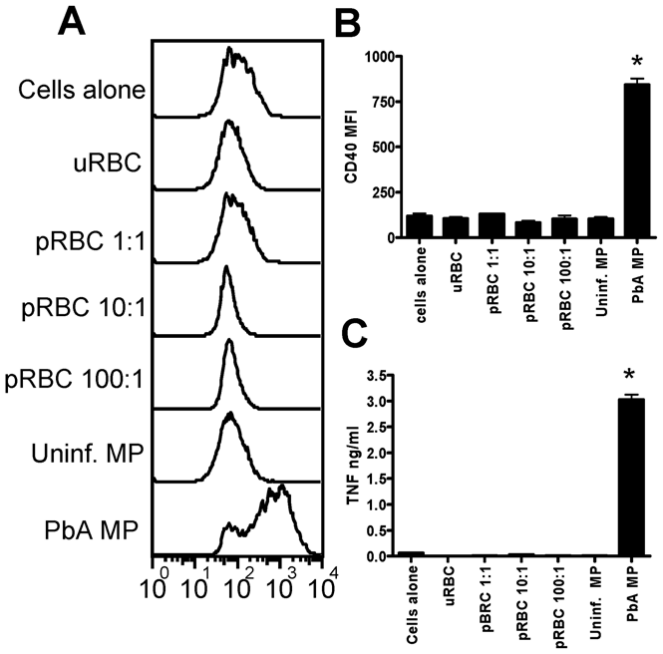
Malaria infection derived plasma microparticles promote significantly stronger macrophage activation than intact parasitized red blood cells. Microparticles were prepared from the plasma of uninfected mice (uninfected MP) and from mice infected with *P. berghei* ANKA (day 7: PbA MP). Mature trophozoite and schizont stage parasitized red blood cells (pRBC) were purified in vitro from blood of PbA infected mice. (A) Representative histograms showing the level of CD40 expression by bone marrow derived macrophages following stimulation with uninfected RBCs, pRBCs and uninfected MPs and PbA MPs. (B) Mean fluorescence intensity of CD40 expression by macrophages following stimulation. (C) The level of TNF production by stimulated macrophages was measured in the supernatant by ELISA. The results are representative of 2 separate experiments. * denotes significant difference between PbA MP and no stim.cultures.

Our results in Figure 1 demonstrated the ability of PbA infection induced MPs to stimulate pro-inflammatory responses *in vitro*, but these results did not specifically address the potential relevance of this pathway in the generation of inflammatory responses during malaria infection. Consequently, to examine the importance of MP-induced macrophage activation during malaria infection, and how this may relate to other parasite-specific pathways of macrophage activation, we compared the ability of infection-induced MPs and live intact PbA pRBC to promote macrophage activation. As expected, stimulation with uninfected red blood cells failed to induce up-regulation of CD40 expression or the production of TNF by macrophages (Fig. 2A–C). Interestingly, stimulation with live intact parasitized red blood cells (>80% purity, mainly late trophozoites and schizonts) at 1∶1, 10∶1 and 100∶1 ratios of parasites to macrophages also failed to induce up-regulation of CD40 expression or the production of TNF (Fig. 2A–C). These results are surprising as strong macrophage activation, including pro-inflammatory cytokine production, has been reported following in vitro stimulation with *P. falciparum* schizont infected RBC [22]. In contrast, although a number of studies have clearly demonstrated that phagocytosis of murine pRBC by macrophages occurs in vitro [36]–[38], there is very little evidence to suggest that this leads to up-regulation of co-stimulatory receptor expression on macrophages, or the production of pro-inflammatory cytokines [39]. Nonetheless, stimulation with infection induced MPs promoted significant up-regulation of CD40 expression and production of TNF by macrophages (Fig. 2A–C). These results show that plasma derived MPs may exert a dominant pathway in driving macrophage activation during malaria infection, either causing much of the inflammation and pathology of infection, or initiating anti-malaria immune responses.

### Analysis of MP phenotype

MPs derived from malaria infected mice were considerably more inflammatory than uninfected MPs derived from naive mice (Fig. 1), even when the numbers of particles in each preparation was normalised, suggesting that MPs derived from malaria infected mice are more immunogenic than uninfected MPs. MPs can be produced by the vesiculation of the membrane of many different cell populations, including platelets, leukocytes, endothelial cells and red blood cells, a process that is modulated during inflammatory episodes [35]. Consequently, the predominant cellular source of the MPs may change during malaria infection, and this alteration in cellular source could explain the difference in ability to promote macrophage activation. To address this likelihood, we performed a phenotypic characterisation of the PbA infection derived and uninfected MP populations.

**Figure 3:**
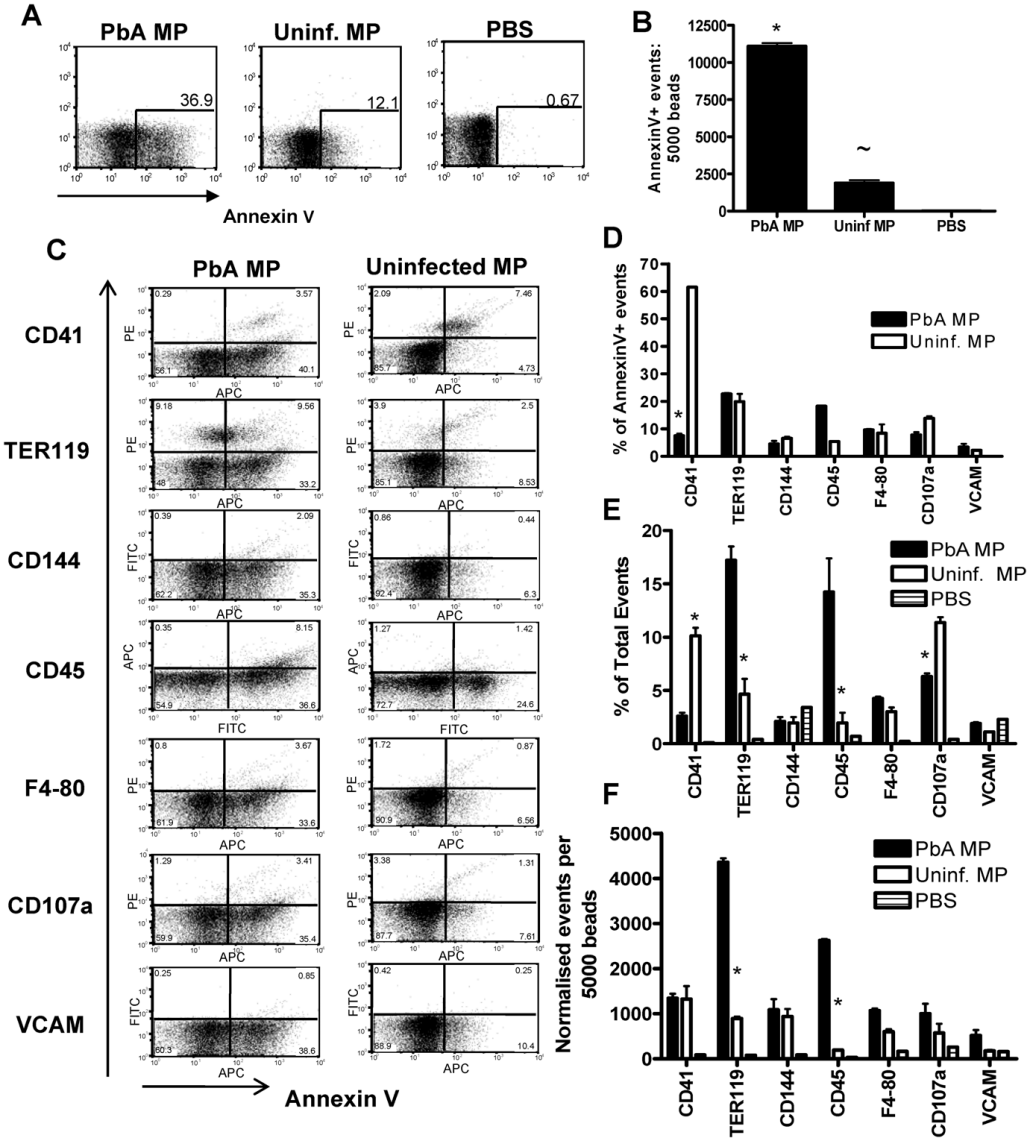
Phenotypic characterization of uninfected and *P. berghei* ANKA plasma derived microparticles. Microparticles were prepared from the plasma of uninfected mice (uninfected MP) and from mice infected with *P. berghei* ANKA (day 7: PbA MP). The cellular sources and composition of the MPs were determined by flow cytometry. (A) Representative dot plots showing the frequency and (B) normalized numbers of Annexin V+ events within the uninfected MP and PbA MP preparation. (C) The cellular source and activation status of MPs were examined by determining the expression of TER119, CD144, CD45, F4-80, CD107a, CD41 and VCAM-1. (D) The percentage of Annexin V positive events co-expressing secondary markers. (E) The frequency and (F) numbers of flow cytometric events in uninfected and PbA MPs expressing TER119, CD144, CD45, F4-80, CD107a, CD41 and VCAM-1. The results are representative of 2 separate experiments. * p<0.05 between PbA MP and uninfected MP; ∼ P<0.05 between uninfected MP and PBS.

We first assessed the expression of Annexin V, a marker of cellular apoptosis, which is the standard marker of classical inflammation-driven microparticles [35]. As expected based on previous reports [33],[40], we observed a significant and marked increase in the expression of Annexin V on PbA induced MPs compared with uninfected MPs, both in terms of frequency and total numbers of positive events (Fig. 3A, B). Very low Annexin V staining was observed on the PBS control FACS events, demonstrating the specificity of the flow cytometric staining (Fig. 3A, B). Importantly, not all infection derived (or uninfected) MPs expressed AnnexinV, indicating that a large proportion of particles within the MP preparation are not classically defined or produced MPs.

To examine the cellular source of AnnexinV+ (and AnnexinV−) microparticles we employed a panel of antibodies to cover all the major potential cellular sources of MPs (Fig. 3C). Platelets (CD41) were the major source of classical AnnexinV+ microparticles within the uninfected MP population, with a number of red blood cell (TER119) derived Annexin V+ MPs also found (Fig. 3C, D). Few AnnexinV+ microparticles within the uninfected MP population were produced from endothelium (CD144), leukocytes (CD45) and macrophages (F4-80) and only a small number co-expressed CD107a, meaning most were not exosomes, or VCAM-1, indicating that they did not emanate from activated endothelium (Fig. 3C, D). In contrast the majority of AnnexinV+ MPs derived from PbA infected mice did not co-express CD41, suggesting that they were not platelet derived. Approximately 20% of PbA infection induced AnnexinV+ microparticles appeared to be derived from red blood cells, but the majority of the AnnexinV+ microparticles failed to co-stain with any tested antibody, meaning that their cellular origin is undefined (Fig 3C, D). Nevertheless, when breaking down the cellular sources of all the flow cytometric events within the PbA infection induced and uninfected MP preparations, irrespective of AnnexinV expression, a clear increase in the frequency and numbers of TER119+ (RBC derived) and CD45+ (leukocyte derived) flow cytometric events was observed within the PbA infection induced MP population (Fig. 3C, E and F).

### Inflammatory malaria infection-induced microparticles are derived from pRBC

**Figure 4:**
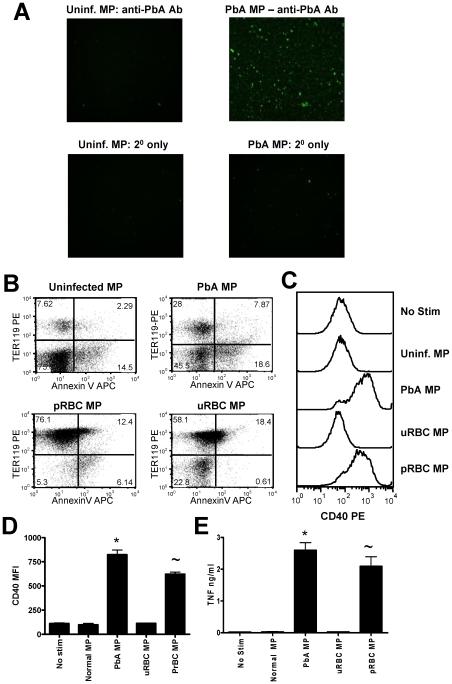
Immunogenic malaria infection-derived MPs are produced from pRBC and contain parasite materials. Microparticles were prepared from the plasma of uninfected mice (uninfected MP) and from mice infected with *P. berghei* ANKA (day 7: PbA MP). (A) The presence of parasite material in MP preparations was examined by IFAT using purified anti-*P. berghei* ANKA IgG antibodies followed by detection with FITC-labelled anti-mouse secondary antibodies. MPs were prepared from purified pRBC (pRBC MP) and the ability of pRBC MP to activate macrophages *in vitro* relative to PbA plasma derived MPs was assessed. (B) The expression of AnnexinV and TER119 on the different MP preparations. (C) Representative histograms showing the level of CD40 expression by bone marrow derived macrophages following stimulation with uninfected MPs, PbA MPs, uninfected RBC MPs (uRBC MPs) and pRBC MPs. (D) Mean fluorescence intensity of CD40 expression by macrophages following stimulation. (E) The level of TNF production by stimulated macrophages was measured in the supernatant by ELISA. The results are representative of 2 separate experiments. * p<0.05 between PbA MP and no stim.cultures; ∼ p<0.05 between pRBC MP and no stim.cultures.

The phenotypic characterisation of PbA infection-induced MPs and uninfected MPs demonstrated clear differences in the expression level of AnnexinV, TER119 and CD45, suggesting that the predominant cellular sources of PbA infection induced MPs and uninfected MPs varied, potentially explaining the inflammatory nature of the malaria infection induced MPs. As the frequency and numbers of RBC-derived particles was increased in the PbA infection induced MP preparation, we hypothesised that infection-induced MPs may either be formed by vesiculation of the pRBC membrane during intra-erythrocytic parasite maturation and/or pRBC rupture at schizogeny, in which case MPs would be expected to contain malaria parasite-derived components, or that they may be derived from uninfected RBCs, which are known to undergo bystander lysis during acute malaria infection, contributing to the rapid onset of anaemia [3]. To distinguish between these two possibilities, we analysed PbA infection-induced MPs for the presence of PbA-specific antigens by immunofluorescence using purified anti-PbA IgG. As expected, no parasite-derived material was detected in uninfected MP preparations (Fig. 4A). In contrast a large proportion of MPs from PbA-infected mice bound the anti-PbA IgG (Fig. 4A), indicating the presence of significant quantities of parasite-derived material in the infection-derived MPs. Although a number of parasite moieties are likely to be incorporated within the malaria infection induced MP preparation, we failed to detect hemozoin in the plasma derived preparation by beta-hematin formation assay (results not shown).

### pRBC-derived microparticles are phenotypically and functionally distinct from inflammation-induced microparticles

**Figure 5:**
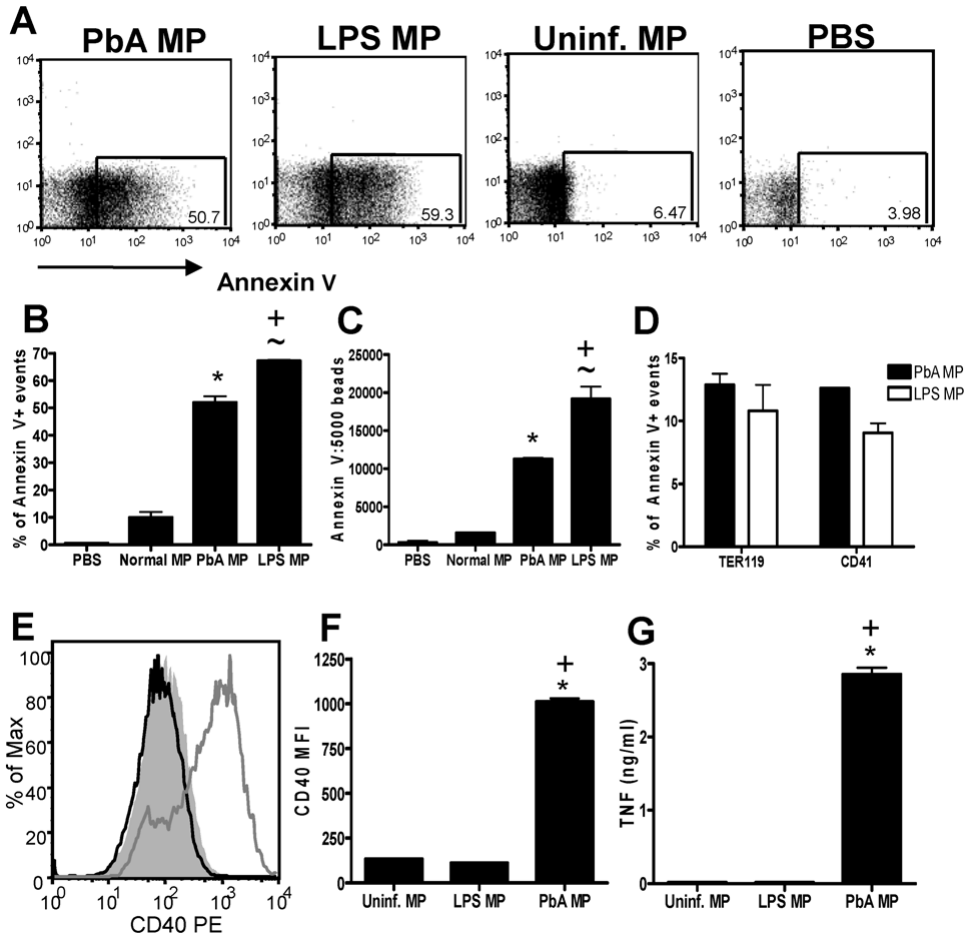
LPS-inflammation induced MPs do not activate macrophages. Microparticles were prepared from the plasma of uninfected mice (uninfected MP), from mice injected with 20µg LPS (Day 3: LPS MP) and from mice infected with *P. berghei* ANKA (day 7: PbA MP). (A) Representative dot plots showing the expression of Annexin V in the MP preparations. (B) The frequency and (C) normalized numbers of Annexin V+ events within the MP preparations. (D) The frequency of TER119+ and CD41+ events within the Annexin V+ populations. (E) Representative histograms showing the level of CD40 expression by bone marrow derived macrophages following stimulation with MPs: shaded histogram no stimulation; dark line LPS MP; light line PbA MP. (F) Mean fluorescence intensity of CD40 expression by macrophages following stimulation. (G) The level of TNF production by stimulated macrophages was measured in the supernatant by ELISA. The results are representative of 2 separate experiments. * p<0.05 between PbA MP and no stim.; ∼ p<0.05 between LPS MP and no stim.; + p<0.05 between PbA MP and LPS MP.

The observation that a large proportion of PbA-infection induced MPs were derived from pRBC and were predominantly Annexin V^−^ suggested that they were not classical inflammation-induced MPs. Nevertheless, PbA-infection induced MPs also displayed heterogenous expression of Annexin V and the frequency and numbers of Annexin V^+^ MPs increased during malaria infection, showing that inflammatory MPs were also produced during infection. Since it is not feasible to efficiently separate the two populations of MPs from the plasma of PbA-infected mice, to determine whether macrophage activation was induced by the pRBC-derived MPs or by the more classical Annexin V^+^ MPs, we generated a pure population of pRBC-derived MPs in vitro from purified and extensively washed pRBC and compared their macrophage activating properties with MPs from the plasma of PbA-infected or uninfected mice, with *in vitro* generated MPs from uRBC (Fig. 4) and with MPs purified from the plasma of mice treated with LPS to induce inflammation (Fig. 5). The numbers of MPs in each preparation were counted by flow cytometry and were normalised to the number of MPs from uninfected mice prior to culture with BMDM.

The *in vitro*-derived MPs were of similar size to those prepared from plasma (data not shown). In vitro-derived MPs were predominantly (>80%) TER119^+^, and although a proportion of the *in vitro*-derived MPs (<20%) expressed Annexin V^+^, the levels of Annexin V expression were substantially lower (MFI of 40.0 for *in vitro*-derived MPs vs MFI of 98.5 for infection-derived MPs) than for infection-derived MPs (Fig. 4B).

In support of our hypothesis that parasite material bound to RBC membranes within the MPs is responsible for macrophage activation, we observed significant up-regulation of CD40 expression (Fig. 4C, D) and production of TNF (Fig. 4E) by macrophages cultured with *in vitro*-derived pRBC MPs, which was comparable to the activation observed with the PbA infection-derived plasma MPs (Fig. 4C–E). In contrast, MPs derived *in vitro* from uRBC did not induce macrophage activation. As the vast majority of pRBC derived MP particles expressed TER119 (Fig. 4B), it is unlikely that soluble non-membrane associated parasite materials were purified during the generation of the MP preparation or that non-membrane bound parasite materials were responsible for the macrophage activation.

The plasma of mice injected 3 days previously with LPS - to induce inflammation - contained MPs that expressed high levels of Annexin V^+^ (Fig. 5A) and did not stain with the anti-PbA antiserum (data not shown). LPS was a more potent stimulus of classical Annexin V^+^ MP generation than PbA infection, leading to an increase in both the frequency (Fig. 5B) and total numbers (Fig. 5C) of Annexin V^+^ MPs. Although fewer Annexin V^+^ MPs were present in the plasma of PbA-infected mice, the phenotype of these Annexin V^+^ MPs was very similar to that of the LPS-induced MPs, based upon TER119 and CD41 co-staining (Fig. 5D), indicating that classical inflammation-induced MPs - derived from comparable cellular sources - were present in both MP preparations. Despite this, the LPS-induced MPs failed to induce macrophage activation whereas, as shown previously, PbA-infection induced MPs induced up-regulation of CD40 expression and production of TNF (Fig. 5E–G).

Taken together, these data demonstrate that classical, inflammation-driven MPs do not directly induce pro-inflammatory immune responses in macrophages but that the atypical MPs generated from pRBC have potent pro-inflammatory activity.

### MP particle generation correlates with the onset of clinical signs

**Figure 6:**
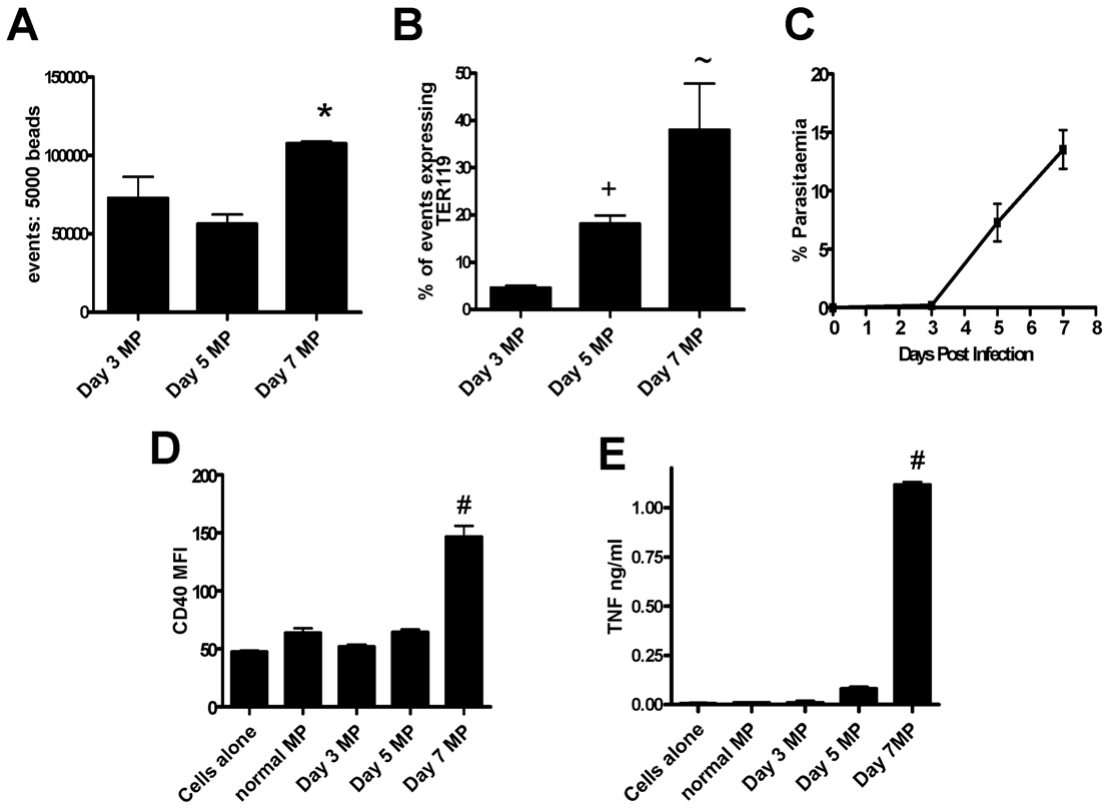
The generation of TER119^+^ immunogenic MPs depends on the stage of malaria infection. Macrophages were stimulated for 24hrs with microparticles prepared from the plasma of mice infected with *P. berghei* ANKA. (A) Mean fluorescence intensity of CD40 expression by macrophages following stimulation. (B) The level of TNF production by stimulated macrophages was measured in the supernatant by ELISA. (C) The number of MPs within the PbA derived preparations was calculated relative to a standardized number of 1µm beads. (D) The expression of TER119 on the different MP preparations. (E) The percentage parasitaemia on days 3, 5 and 7 post-infection. The results are representative of 2 separate experiments. * p<0.05 between day 5 PbA MP and day 7 PbA MP; + p<0.05 between day 3 PbA MP and day 5 PbA MP; ∼ p<0.05 between day 3 PbA MP and day 7 PbA MP; # p<0.05 between day 7 PbA MP and all groups.

To determine whether the timing of inflammatory MP generation correlated with the onset of clinical signs, the numbers and the inflammatory potential of MPs isolated from mice on days 3, 5 and 7 of infection were compared (Fig. 6). Although the total number of plasma MPs increased only slightly over the course of infection (Fig. 6A), numbers of TER119^+^ erythrocyte-derived MPs increased steadily over the course of infection (Fig. 6B) in line with the steadily increasing parasitaemia (Fig. 6C). Importantly, however, day 3 and day 5 MPs were only poorly pro-inflammatory (Fig. 6D,E) and only day 7 MPs were able to induce significant macrophage CD40 expression and TNF production, suggesting either that there is a threshold concentration of MPs required for macrophage activation or that day 7 MPs are qualitatively different from day 3 or day 5 MPs. In either event, there is clearly a close temporal association between the accumulation of highly pro-inflammatory MPs and the onset of severe malarial disease.

### Inflammation is not required for production of immunogenic microparticles during malaria infection

**Figure 7:**
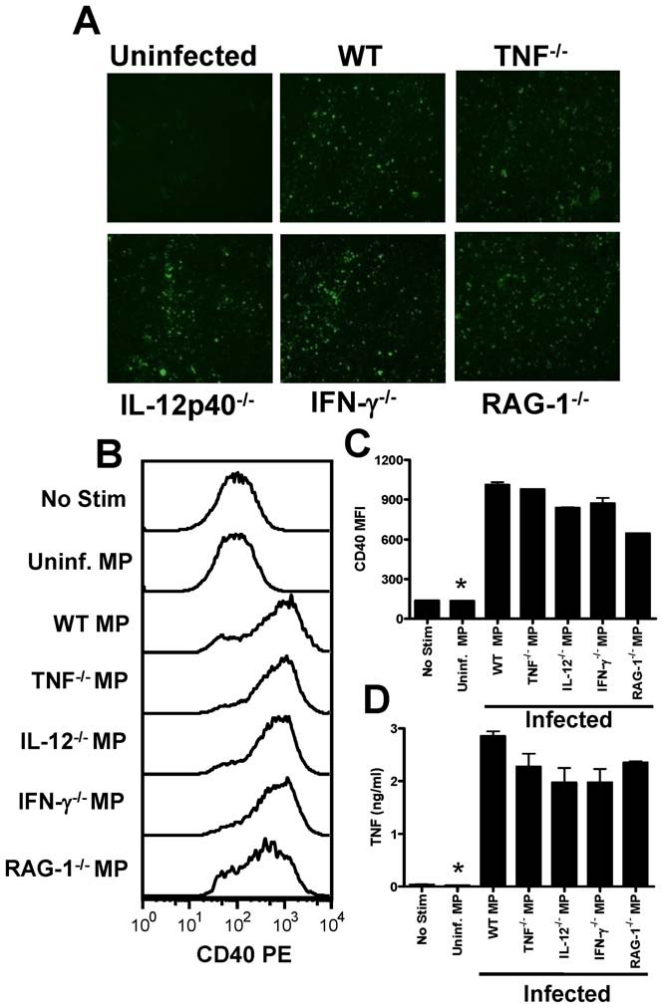
Microparticles generated during malaria infection independently from inflammation promote macrophage activation. Microparticles were prepared from the plasma of uninfected mice (uninfected MP) and from WT, TNF^−/−^, IL-12p40^−/−^, IFN-γ^−/−^ and RAG-1^−/−^ mice infected with *P. berghei* ANKA (day 7: PbA MP). (A) The presence of parasite material in the separate MP preparations was examined by IFAT. (B) Representative histograms showing the level of CD40 expression by bone marrow derived macrophages following stimulation with MPs. (C) Mean fluorescence intensity of CD40 expression by macrophages following stimulation. (D) The level of TNF production by stimulated macrophages was measured in the supernatant by ELISA. The results are representative of 2 separate experiments. * denotes significant difference between no stim and all infection derived MP preparations.

We have shown that pRBC-derived microparticles are phenotypically distinct from classical, inflammation-induced microparticles and, in contrast to LPS-induced MPs, possess potent pro-inflammatory activity. Nonetheless, AnnexinV^+^ inflammation-driven MPs have been reported to induce pro-inflammatory immune responses in other models [35]. Thus, to further explore the role of inflammation and inflammation-induced MPs in the response of macrophages to PbA-induced MPs, we prepared MPs from the plasma of PbA-infected (day 7 p.i.) TNF^−/−^, IL-12p40^−/−^, IFN-γ^−/−^ and RAG-1^−/−^ mice, all of which have major defects in their innate inflammatory response, and compared them with MPs from WT PbA-infected mice (Fig. 7). Numbers of MPs in each preparation were counted (by flow cytometry) and adjusted to uninfected MP numbers. Similar levels of immunofluorescence were seen when MPs were labelled with anti-PbA antiserum (Fig 7A), indicating that comparable amounts of parasite material was found in each preparation. Importantly, MPs derived from PbA-infected TNF^−/−^, IL-12p40^−/−^, IFN-γ^−/−^, RAG-1^−/−^ and WT mice all induced very similar levels of macrophage activation, as shown by up-regulation of CD40 expression and TNF production (Fig. 7 B–D). Taken together, these data strongly suggest that parasite moieties within the membranes of these atypical, pRBC-derived MPs are responsible for macrophage activation and that these MPs can be generated in the absence of inflammation.

### Microparticle driven macrophage activation is TLR4 and MyD88 dependent

**Figure 8:**
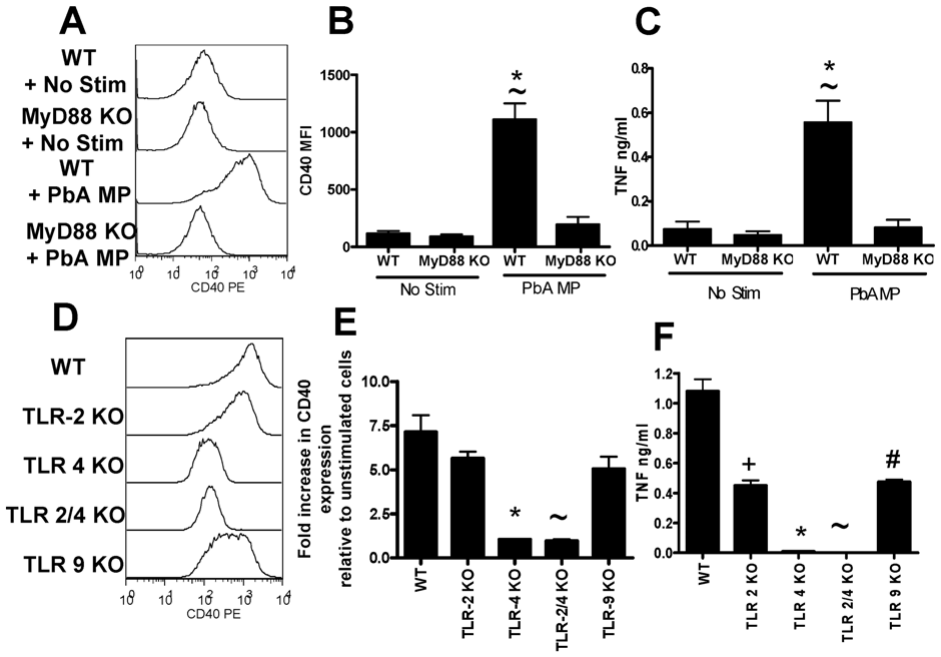
Microparticle driven macrophage activation is MyD88 and TLR-4 dependent. Microparticles were prepared from the plasma of mice infected with *P. berghei* ANKA (day 7: PbA MP). Bone marrow derived macrophages were generated from WT (B6), MyD88^−/−^, TLR-2^−/−^, TLR-4^−/−^, TLR-2/4^−/−^ and TLR-9^−/−^ mice. The ability of PbA MPs to activate (A–C) MyD88^−/−^ macrophages and (D–F) TLR-2^−/−^, TLR-4^−/−^, TLR 2/4^−/−^ and TLR-9^−/−^ macrophages was examined. (A, D) Representative histograms showing the level of CD40 expression by macrophages following stimulation with PbA MPs. (B) Mean fluorescence intensity of CD40 expression by macrophages following stimulation. (E) normalized CD40 expression by macrophages relative to non-stimulated controls. (C, F) The level of TNF production by stimulated macrophages was measured by ELISA. The results are representative of 2 separate experiments. (B, C) * p<0.05 between WT non-stimulated and PbA MP stimulated; ∼ p<0.05 between WT PbA MP stimulated and MyD88^−/−^ PbA MP stimulated. (E, F) * p<0.05 between WT PbA MP stimulated and TLR-4^−/−^ PbA MP stimulated; ∼ p<0.05 between WT PbA MP stimulated and TLR-2/4^−/−^ PbA MP stimulated; + p<0.05 between WT PbA MP stimulated and TLR-2^−/−^ PbA MP stimulated; # p<0.05 between WT PbA MP stimulated and TLR-9^−/−^ PbA MP stimulated.

We next investigated the pathways required for macrophage activation by malaria infection induced MPs. As our data indicated that parasite material, bound to RBC membrane within the MP preparation was responsible for driving macrophage activation, we hypothesised that TLRs and the adaptor molecule MyD88 may be required for macrophage stimulation. TLR molecules and MyD88 have previously been shown to be required for optimal pro-inflammatory cytokine production during malaria infection [10],[12],[17],[20]. Our results clearly show that PbA infection-induced MP activation of macrophages is MyD88 dependent, as macrophage activation, as measured by CD40 up-regulation and TNF production, was completely ablated in MyD88^−/−^ macrophages (Fig. 8A–C). MP-induced macrophage activation was totally TLR-4 dependent; up-regulation of CD40 expression and induction of TNF production were both completely absent in TLR-4^−/−^ macrophages (Fig. 8D–F). Interestingly, however, PbA infection-induced MP stimulation of TNF production (but not CD40 expression) was also significantly lower in TLR-2^−/−^ and TLR-9^−/−^ macrophages than in WT macrophages. These data would be consistent with the presence of low levels of TLR-2 and TLR-9 ligands such as GPI and hemozoin within the MPs. As the response was completely ablated in TLR-4^−/−^ macrophages - demonstrating that TLR-4 is essential for TLR-4 responsiveness - our results suggest that a primary TLR-4 ligand within the MPs activates the cells and that TLR-2 and TLR-9 ligands synergise with the TLR-4 stimulus to induce maximal macrophage activation.

## Discussion

Understanding the pathways leading to inflammation during malaria infections should allow the development of new approaches to therapy and more immunogenic vaccines. In this study we have identified an entirely novel pathway of inflammation during malaria infection, namely TLR-4/MyD88-mediated activation of macrophages by membrane microparticles emanating from parasitized red blood cells. Importantly, we have shown that malaria-infection induced MPs promote significantly stronger macrophage activation than live infected red blood cells, underlining the potential significance of MP-induced inflammation during malaria infection. The primary TLR-4 dependence of this pathway sets it apart from the previously described glycoslylphosphatidylinositol (GPI)-TLR-2/CD36 pathway [19]–[20] and the hemozoin/parasite DNA/TLR-9 pathway [12].

Previous studies on MPs during malaria infection have largely been in the context of their role in the pathogenesis of severe disease: circumstantial evidence supports a role for MPs in severe *P. falciparum* infection [36]. In addition, ABCA1 KO mice that are defective in the ability to produce MPs are protected against ECM during *P. berghei* ANKA infection [33]. Endothelial and platelet derived MPs have been shown to “bridge” endothelial cell and pRBC and leukocyte interactions, allowing sequestration within brain microvessels, which is a key factor in initiation of cerebral pathology [41],[42]. However, other than the studies in ABCA1 KO mice, which have numerous defects in lipid metablism and macrophage function that might influence their susceptibility to ECM [43], there is no causal evidence to link MPs with pathogenesis in vivo. The results of this current study have added to the complexity of the potential roles for MPs during malaria infection. We have shown that malaria infection induced MPs are capable of promoting the up-regulation of CD40 expression and TNF secretion from bone marrow derived macrophages in vitro; thus, malaria infection induced MPs promote potent activation of innate and adaptive immune responses, which is likely to have major significance in the development of inflammation during infection.

Our data importantly differs from earlier studies on MPs during malaria infection, as we have defined the cellular source of immunogenic MPs as infected red blood cells, containing large quantities of parasite-material, rather than platelets or endothelial cells [36], [40], [40]–[42]. pRBC-derived MPs were phenotypically and functionally distinct from the classical, Annexin V^+^ microparticles that emanate primarily from platelets but also from leucocytes and endothelial cells during systemic inflammation [34],[35]. Not only were pRBC-derived MPs produced in the absence of key inflammatory mediators such as TNF, IL-2 and IFN-γ but also, in our hands, classical inflammation-derived MPs had minimal macrophage activating capacity and are thus clearly products of - rather than drivers of - inflammation.

One particularly striking observation was that pRBC-derived MPs are much, much more potent macrophage activators than are live, intact pRBC. This suggests that MPs may be released from pRBC only at certain very specific stages of parasite development, possibly associated with the membrane disintegration that is seen immediately prior to schizont rupture [44]. Although mature pRBC are expected to rupture and produce MPs during the course of the 24hr culture, the concentration of MPs generated may have been too low to activate the macrophages. Alternatively, phagocytosis of intact pRBC by macrophages via CD36-dependent pathways [39] may have prevented generation of free microparticles or suppressed the subsequent TLR-4 mediated signalling promoted by MPs. In agreement with the latter hypothesis, macrophage activation through anti-CD40 or TLR stimulation is suppressed following the phagocytosis of non-activating latex beads [45] and TLR-tolerance has been shown to occur during malaria infection [46].

The importance of parasite material within the MPs for stimulating macrophage activation raises intriguing questions regarding the nature and identity of the pro-inflammatory parasite molecules within the MPs. GPI, the membrane anchor for MSP-1 and MSP-2, and hemozoin, the product of haemoglobin breakdown by the parasite, have been shown to promote [12],[19],[20],[31] or suppress innate activation [28]–[30]. While hemozoin is thought to stimulate macrophage activation through ligation with TLR-9 [12],[27], activation by GPI is mediated by TLR-2 and CD36 through downstream activation of ERK, p38, MAPK, JNK and NFκb signalling pathways [19],[20],[47],[48]. The absolute dependence of MP-mediated macrophage activation on TLR-4 signalling makes it most unlikely that MPs are simply vehicles for GPI and hemozoin. Furthermore, plasma-derived PbA infection-induced MPs did not contain measurable levels of hemozoin and we have found that scavenger receptor A and B family members (including CD36) are not required for MP-induced macrophage activation (results not shown). These data suggest that the main inflammatory parasite materials within the MP preparation are unlikely to be GPI or hemozoin. Nevertheless, TLR-2 KO and TLR-9 KO macrophages produced significantly lower levels of TNF following stimulation with PbA infection-induced MPs, indicating that TLR-2 and TLR-9 signalling is required for optimal TNF production. These observations are highly consistent with a scenario in which PbA infection-induced MP recognition by TLR-4 initiates the macrophage response and macrophage activation is then amplified by GPI/hemozoin signalling through TLR-2 and TLR-9. Co-operation and synergy of distinct TLR signalling pathways in response to complex TLR ligands is becomingly increasingly well recognised in a number of different systems [49],[50].

The essential role for MyD88 signalling for macrophage activation by malaria-infection derived MPs is entirely consistent with a number of studies demonstrating a role for MyD88 in malarial inflammation and pathology. For example TLR/MyD88 mediated IL-12 production is responsible for liver injury during *P. berghei* NK65 infection [9] and TNF production induced via MyD88 signalling promotes weight loss and fever during *P. chabaudi* AS infection [17]. Moreover, although the role of MyD88 dependent signalling in *P. berghei* ANKA induced ECM remains unclear with conflicting findings [10],[11],[13],[14], MyD88 signalling is required for optimal macrophage TNF, IL-6 and IL-1α production [10]. Our data suggest that pRBC-derived MPs may be a significant inducer of all these effects.

In conclusion, we have identified a novel and very potent, MyD88- and TLR-4-dependent pathway of inflammation during malaria infection that is mediated by pRBC-derived membrane microparticles. Interestingly, a recent study has shown a link between polymorphisms in the *TLR-4* locus and susceptibility to severe malaria in humans [51]; our data offer a plausible biological explanation for this observation. We expect that pRBC microparticles will synergise with GPI and hemozoin to induce the extraordinarily high levels of circulating inflammatory mediators that are seen in many patients with acute malaria. However, the ability of MPs to induce expression of molecules such as CD40 on antigen presenting cells suggests that they might also play a role in T cell priming and T effector cell function. Future studies will need to identify the parasite ligands presented by these microparticles and explore their potential role as adjuvants for malaria vaccines.

## Materials and Methods

### Ethics statement

Animal experimentation was approved under UK Home Office Regulations and was subject to LSHTM ethical review.

### Mice and parasites

Female, 8–12 week old C57BL/6 wild type, IFN-γ^−/−^, TNF^−/−^, IL-12^−/−^, and RAG-1^−/−^, were obtained from Harlan and maintained under barrier conditions.

Cryopreserved *Plasmodium berghei* ANKA parasites were passaged once *in vivo* for a maximum of 4 days before being used to infect experimental animals. Mice were infected intraveneously with 10^4^ parasitised red blood cells and parasitaemia was determined daily by examination of Giemsa-stained thin blood smears.

### Preparation of plasma microparticles

MPs were prepared as described before [33]. Briefly, blood was collected aseptically in 0.124M sodium citrate solution from naive or malaria-infected mice (day 6 or 7 of *P. berghei* ANKA infection), and centrifuged at 1,500g for 15 minutes at room temperature. The platelet poor plasma supernatant (PPP) was collected and further centrifuged at 13,000g for 3mins to obtain platelet free plasma (PFP). This was diluted 1∶3 with citrated PBS containing heparin and centrifuged at 14,000g for 90 minutes at 15°C to produce the MP pellet which was then resuspended in sterile PBS. MPs were quantified by flow cytometry as numbers of events relative to a standardised number of 1µm beads (BS Partikels, GMBH, Germany). Unless otherwise stated, blood from two mice was pooled to generate each MP preparation. All microparticle preparations and negative control samples were tested for LPS contamination using the Limulus Amebocyte Lysate (LAL) gel formation test, performed according to manufacturer's standard operating procedures (Health Protection Agency, UK).

### Erythrocyte-derived microparticles

*P. berghei* ANKA pRBC were collected on day 6 or day 7 of infection and enriched using LD column magnetic cell sorting (Miltenyi Biotec). pRBC were routinely >80% pure and were mainly mid- to late-stage trophozoites and schizonts. pRBC were washed three times in PBS to remove all plasma constituents and the final pellet was resuspended to the original volume. To make microparticles, pRBC or equivalent numbers of RBC from uninfected mice (uRBC) were subject to repeated (3X) combinations of freeze-thaw and ultra-sonication (10 sec/pulse) cycles. pRBC or uRBC lysates were then centrifuged at 13,000g for 3min to remove particulate material and the supernatant was diluted 1∶3 with citrated PBS containing heparin and centrifuged at 14,000g for 90 minutes at 15°C to produce the MP pellet. All microparticle preparations and negative control samples were tested for LPS contamination using the Limulus Amebocyte Lysate (LAL) gel formation test, performed according to manufacturer's standard operating procedures (Health Protection Agency, UK).

### Bone marrow derived macrophages (BMDM)

Bone marrow derived macrophages harvested from femurs of wild type and knockout mice were prepared as described previously [52]. Briefly, bone marrow cells were washed and suspended in DMEM supplemented with 10% heat-inactivated FCS, 20% L-cell supernatant (a source of CSF-1), 5% horse serum (SIGMA), L-glutamine (GIBCO) and penicillin and streptomycin (GIBCO) and cultured in tissue culture Petri Dishes (Sterilin, UK). After 7 days the supernatant containing fibroblasts and mature macrophages was removed. Adherent cells were scraped off gently, washed, diluted 1∶3 and cultured to maturity for a further 4 days. Macrophages were cryo-preserved until required.

### Macrophage activation assay

BMDM were cultured at 10^6^/ml in duplicate or triplicate in 96-well plates (NUNC) either in DMEM alone or with LPS (200ng/nl), pRBC/uRBC (at various BMDM∶RBC ratios) or MPs (normalised numbers in each experiment relative to uninfected MP levels: average 3–5×10^5^/well, depending on the experiment) for 24 hours at 37°C in 5% CO_2_). Supernatants were collected and assayed for TNF and the macrophage monolayer was harvested for flow cytometric analysis.

### ELISA

Supernatants were assayed by standard capture ELISA using Immunolon 4 HBX plates (ThermoLabsystems UK) coated with monoclonal hamster antibody against murine TNF (TN3) (gift from Celltech, Slough, UK). Bound TNF was detected with a biotinylated goat anti-mouse TNF-α antibody (R&D, UK), streptavidin peroxidase (Sigma, UK) and *0*-phenylenediamine (Sigma, UK). Recombinant murine TNF (R&D, UK) was used as a standard.

### Flow cytometry

MPs were quantified relative to a standardised number of 1µm reference beads (BS Partikels, GMBH, Germany): 5µl of MPs were diluted in 200µl of sterile PBS, with 5 drops of 1µm beads added into 4mls PBS. Phenotypic characterisation of MPs was performed by incubating 10µl MPs, diluted 1∶4 in sterile PBS, with anti-mouse TER119 (clone: TER119), anti-mouse CD41 (clone: MWReg30; BD Pharmingen), anti-mouse VCAM (clone: 429), anti-mouse CD45 (clone: 30F11), anti-mouse CD144 (clone: eBioBV13), anti-mouse CD107a (clone: eBio D4B) or anti-mouse F4-80 (clone: BM8 ). All antibodies, unless otherwise stated, were from E-Bioscience (distributed by Insight Biotechnology, UK). MPs were then suspended in Annexin V (BD Pharmingen) binding buffer before being co-stained with Annexin V. MPs were then diluted to a final volume of 250µl by the addition of sterile PBS containing 1µm reference beads. Macrophage activation was examined by surface staining using anti-mouse CD40 (clone: 1C10), anti-mouse MHC-II (clone: M5/114.15.2) and anti-mouse F4-80 (clone: BM8). Note, significantly altered acquisition settings were applied for flow cytometric detection of MPs compared with those usually utilised for detection of leucocytes; FSc voltage on E02 was adjusted to position 1µm reference beads in the upper right hand area of the plot.

### Indirect Fluorescent Antibody Test (IFAT)

Antiserum to *P. berghei* ANKA-infected RBC was prepared from mice that had undergone 3 rounds of infection and drug cure and IgG was purified on Protein-G (HiTrap, Amersham,UK). Ten microlitres of purified MPs were air dried and acetone fixed on gelatin-coated glass slides. Slides were blocked with rat serum prior to incubation with anti-PbA IgG for 1 hr at room temperature. Following incubation with anti-PbA IgG, slides were visualised using FITC rat anti-mouse antibody (clone 11-4011-85: E-Bioscience) by fluoresescence microscopy (Zeiss, Axioplan 2) using Volocity software (Improvision).

### Scanning electron microscopy

Microparticles were purified from naïve or day 7 *P. berghei* ANKA infected mice as described above. After the final centrifugation at 14,000g for 90min the pellet was resuspended in 30 µl of PBS and MPs were seeded on to pre-coated Poly-L-Lysine-coated glass coverslips (Poly-L-Lysine, Sigma) and allowed to adhere overnight at 4°C in a moist chamber. The adhered MPs were fixed with 30 µl of 2.5% glutaraldehyde and 30 µl of 4% paraformaldehyde for 20 min at room temperature. The 60 µl of fixatives were removed and replaced with fresh solutions of 2.5% glutaraldehyde and 4% paraformaldehyde and left overnight at 4°C in the moist chamber. The coverslips were then washed twice in PBS 0.2 M and left at 4°C until processing. Post-fixation was carried out with osmium for 1h at room temperature and the preparations were dehydrated in solutions of ethanol of increasing strength from 70%, 80% and 90% for 10 min in each solution. Coverslips were finally dehydrated twice for 10min in 100% ethanol and then twice for 5min with 100% ultra-pure ethanol. After rapidly immersing for 3 min in hexamethyldisilazane (Sigma), the preparations were air dried and transferred to a desiccator overnight. Samples were mounted onto, gold coated stubs for 2 min for imaging on a Zeiss scanning electron microscope (Zeiss ULTRA *plus*).

### Statistical significance

Statistical significance was determined using two tailed Student's T test, unless otherwise stated, with P<0.05 considered as significant.

## Supporting Information

Figure S1 Distinct kinetics of CD40 upregulation and TNF production by macrophages following stimulation with LPS and PbA-induced MPs. Macrophages were stimulated for 6hrs, 12hrs, 24hrs or 48hrs with LPS (200ng/ml) or MPs prepared from the plasma of mice infected with *P. berghei* ANKA (day 7: PbA MP). (A) Mean fluorescence intensity of CD40 expression by macrophages following stimulation. (B) The level of TNF production by stimulated macrophages was measured in the supernatant by ELISA. The results are representative of 2 separate experiments.
(0.02 MB PDF)
