# Supplementary figures and images for: Parasite-Derived Plasma Microparticles Contribute Significantly to Malaria Infection-Induced Inflammation through Potent Macrophage Stimulation
Source: PLoS Pathog. 2010 Jan 29;6(1):e1000744. doi: 10.1371/journal.ppat.1000744 (PMC2813278; doi:10.1371/journal.ppat.1000744)

**A**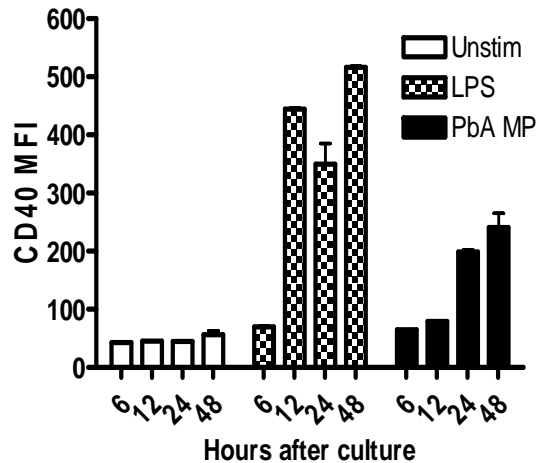**B**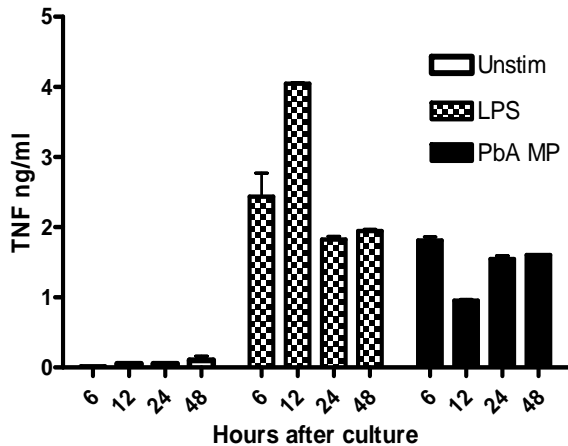

Supplement: Figure S1 — Distinct kinetics of CD40 upregulation and TNF production by macrophages following stimulation with LPS and PbA-induced MPs. Macrophages were stimulated for 6hrs, 12hrs, 24hrs or 48hrs with LPS (200ng/ml) or MPs prepared from the plasma of mice infected with P. berghei ANKA (day 7: PbA MP). (A) Mean fluorescence intensity of CD40 expression by macrophages following stimulation. (B) The level of TNF production by stimulated macrophages was measured in the supernatant by ELISA. The results are representative of 2 separate experiments. (0.02 MB PDF) [file ppat.1000744.s001.pdf]
